# Concurrent heart rate validity of wearable technology devices during trail running

**DOI:** 10.1371/journal.pone.0238569

**Published:** 2020-08-31

**Authors:** James W. Navalta, Jeffrey Montes, Nathaniel G. Bodell, Robert W. Salatto, Jacob W. Manning, Mark DeBeliso

**Affiliations:** 1 Department of Kinesiology and Nutrition Sciences, University of Nevada, Las Vegas, Las Vegas, Nevada, United States of America; 2 Department of Kinesiology, Monmouth College, Monmouth, Illinois, United States of America; 3 Department of Kinesiology, California State University, San Bernadino, San Bernadino, California, United States of America; 4 Department of Kinesiology and Outdoor Recreation, Southern Utah University, Cedar City, Utah, United States of America; Universidade Federal de Mato Grosso do Sul, BRAZIL

## Abstract

Validation of heart rate responses in wearable technology devices is generally composed of laboratory-based protocols that are steady state in nature and as a result, high accuracy measures are returned. However, there is a need to understand device validity in applied settings that include varied intensities of exercise. The purpose was to determine concurrent heart rate validity during trail running. Twenty-one healthy participants volunteered (female n = 10, [mean (SD)]: age = 31 [11] years, height = 173.0 [7] cm, mass = 75.6 [13] kg). Participants were outfitted with wearable technology devices (Garmin Fenix 5 wristwatch, Jabra Elite Sport earbuds, Motiv ring, Scosche Rhythm+ forearm band, Suunto Spartan Sport watch with accompanying chest strap) and completed a self-paced 3.22 km trail run while concurrently wearing a criterion heart rate strap (Polar H7 heart rate monitor). The trail runs were out-and-back with the first 1.61 km in an uphill direction, and the 1.61 return being downhill in nature. Validity was determined through three methods: Mean Absolute Percent Error (MAPE), Bland-Altman Limits of Agreement (LOA), and Lin’s Concordance Coefficient (r_C_). Validity measures overall are as follows: Garmin Fenix 5 (MAPE = 13%, LOA = -32 to 162, r_C_ = 0.32), Jabra Elite Sport (MAPE = 23%, LOA = -464 to 503, r_C_ = 0.38), Motiv ring (MAPE = 16%, LOA = -52 to 96, r_C_ = 0.29), Scosche Rhythm+ (MAPE = 6%, LOA = -114 to 120, r_C_ = 0.79), Suunto Spartan Sport (MAPE = 2%, LOA = -62 to 61, r_C_ = 0.96). All photoplethysmography-based (PPG) devices displayed poor heart rate agreement during variable intensity trail running. Until technological advances occur in PPG-based devices allowing for acceptable agreement, heart rate in outdoor environments should be obtained using an ECG-based chest strap that can be connected to a wristwatch or other comparable receiver.

## Introduction

The use of wearable technology has steadily increased, and has been the top fitness trend since 2016 as determined by health and fitness professionals throughout the world [1]. Wearable devices can return a variety of physiological and health-related metrics including estimates of energy expenditure [2], step count [3], and heart rate [4]. With over 10,000 device options from all ends of the budgetary spectrum [5], determining an appropriate wearable device can be difficult. When considering data output from wearable devices, heart rate is generally the most valid, energy expenditure has been shown to be the least accurate, and step count validity falls between [6]. Focusing on heart rate validity while running, the majority of literature has utilized laboratory-based protocols that are steady state in nature.

Wearable technology devices that return heart rate include smart bras [7], earbuds [8, 9] and sensors placed around the forearm [10, 11] or wrist [12, 13]. Several investigations have utilized treadmill running protocols when evaluating the agreement of heart rate compared with electrocardiogram (ECG) [4, 11, 14, 15] or chest strap criterion measures [8, 10, 16–18], which will be taken as validity in the current investigation. These running protocols have utilized 1.5-minute [11], 3-minute [14, 15, 18, 19], 4-minute [17], 5-minute [4, 8, 10], and 6-minute time frames [16] with a constant speed. In investigations utilizing more than one speed [4, 8, 10, 14, 15, 17–19], treadmill speed at each successive stage is held constant. Reported heart rate validity measures are often combined with walking [8, 10, 11, 19] and are generally high across device types including Jabra ear buds (mean absolute percent error [MAPE] = 2.5%, intraclass correlation coefficient [ICC] = 0.94) [8], Garmin Forerunner 235 (mean difference = 0.1 bpm, ICC = 0.905) [18], Apple Watch (absolute percent difference = 5%, r = 0.93) [11], Fitbit Charge HR (bias = -3 bpm, r = 0.95) [4], and Mio Alpha (mean difference = -4, ICC = 0.91) [14]. As these investigations employed one or more intensities eliciting steady-state heart rate responses, it is likely that the observed high validity measures for heart rate during treadmill running is due to the controlled nature of this activity (sagittal plane motion within a narrow range of motion).

A limited number of studies have not utilized the treadmill, but engaged participants in unrestricted activities including walking and running [13, 20–22]. Dondzlia et al. evaluated the Fitbit Charge HR and Mio FUSE devices in activities including treadmill walking and running and during free living activities for a 24h period [20]. Unfortunately, heart rate validity was not reported for the free living time frame [20]. Reddy et al. utilized the Garmin VivoSmart HR+ and the Fitbit Charge 2 in a 28-min protocol of activities of daily living and interpreted acceptable heart rate accuracy for both devices (mean absolute error = 13%, r = 0.69; mean absolute error = 11%, 0.73 respectively) [21]. Brazendale et al. evaluated the Fitbit Charge HR during a 2h free-living segment in children and determined that it returned accurate heart rate values (absolute percent difference = 7%, r = 0.84) [13]. Montes et al. compared unrestricted walking and jogging through a flat hallway to a speed matched treadmill protocol and found that heart rate was significantly different between environments for certain devices [22]. It was suggested that the differences between environments could at least partially be due to the fact that running speed can vary with free motion activity while it is held constant on the treadmill [22].

As seen from the literature above, agreement between wearable devices and a heart rate criterion measure during running are generally higher during treadmill-based activity [4, 8, 11, 18] and tend to be lower when unrestricted activities are included [13, 21]. It has been suggested that the main source of error with heart rate obtained from photoplethysmography-reliant (PPG) wearable devices is motion artifact in the wave form that decreases accuracy as exercise intensity increases [23]. As running outdoors can be a variable and intermittent activity including high intensity exercise, it is important to determine the accuracy of heart rate obtained from now pervasive wearable technology devices in this applied setting. As our laboratory group has experience obtaining measurements in a variety of natural settings [3, 24–29], and as trail running is increasing in popularity [30, 31], we designed this investigation to determine heart rate validity of wearable monitors during a trail run. It was hypothesized that all devices would display acceptable heart rate validity when compared to a concurrently measured criterion. In order to be acceptable, all of the following criteria must be met: MAPE ≤5%, Lin’s Concordance Coefficient ≥ 0.90, and ICC ≥ 0.70.

## Methods

### Participants

Twenty-one healthy participants volunteered for this study (female n = 10, not presenting with cardiovascular, metabolic, or renal disease; no signs or symptoms suggestive of cardiovascular, metabolic, or renal disease) [32]. Heart rate validity literature has returned large effect sizes (0.91 to 0.95) [4, 18] however, to be conservative a moderate effect size (0.5) was utilized to determine that a total sample size of 21 would be sufficient. Participant descriptive characteristics included the following (mean±SD): age = 31±11 years, height = 173.0±6.9 cm, mass = 75.6±12.9 kg. Participants completed an informed consent document that was approved by the University of Nevada, Las Vegas Biomedical Sciences Institutional Review Board (protocol #1292334).

### Protocol

**Fig 1:**
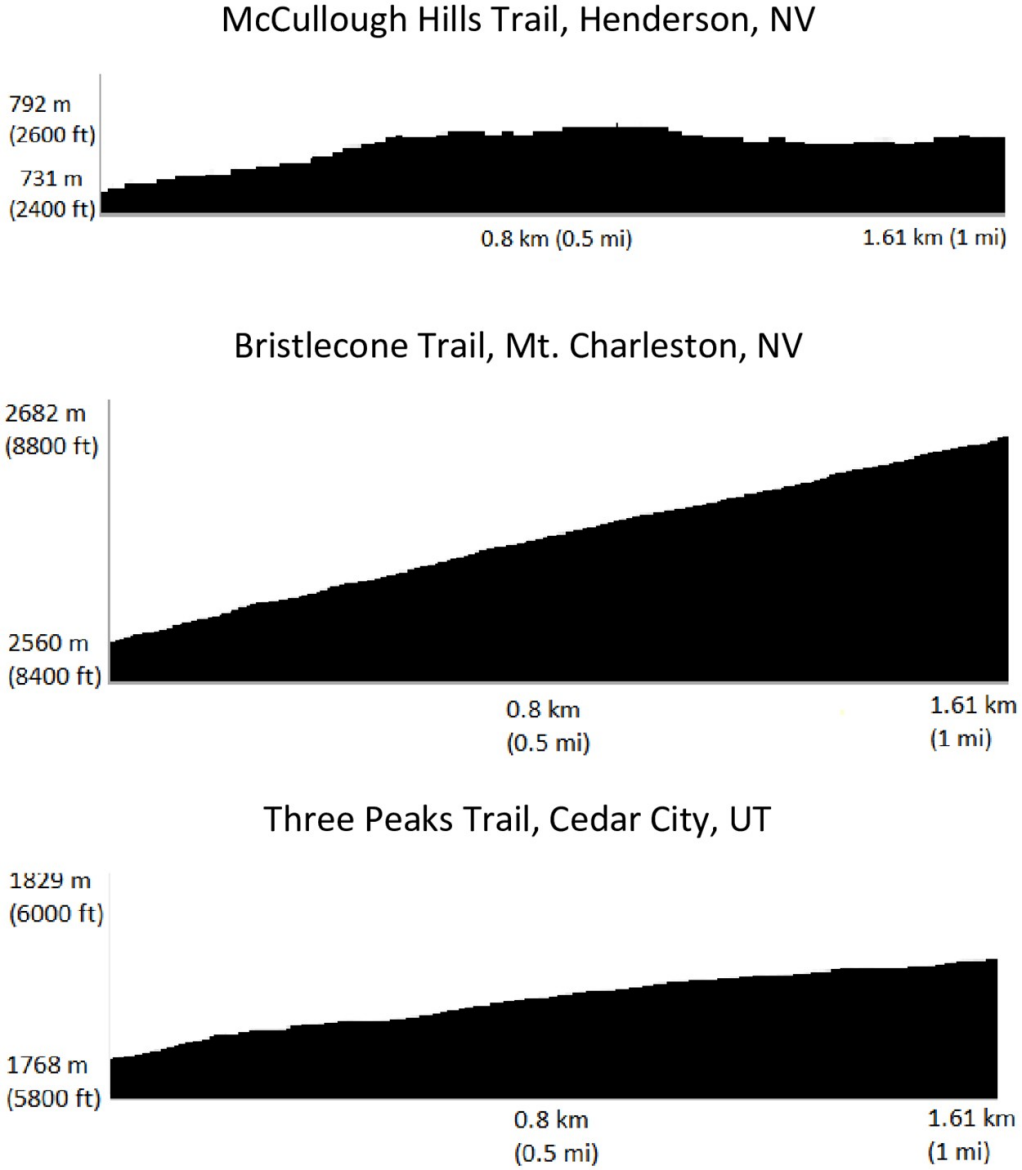
Elevation profiles of the trails utilized by participants (N = 21) outfitted with heart rate returning wearable devices (Garmin Fenix 5 wrist watch, Jabra Elite Sport earbuds, Motiv ring, Scosche Rhythm+ forearm band, and Suunto Spartan Sport watch with accompanying chest strap) concurrently with a criterion device (Polar H7 heart rate monitor).

Participants were outfitted with wearable technology devices and completed a self-paced 3.22 km (two-mile) trail run while concurrently wearing a criterion heart rate strap. Devices were secured to the participant by members of the research team to ensure appropriate fit, and to certify heart rate was obtained by each device. The trail runs were an out-and-back course with the first 1.61 km (1-mile) in a generally uphill direction, and the 1.61 (1-mile) return being generally downhill in nature. Three different trails were utilized which included elevation changes of 48 m (McCullough Hills Trail, Henderson, NV), 55m (Three Peaks Trail, Cedar City, UT) and 104m (Bristlecone Trail, Mt. Charleston, NV). The elevation profiles of these trails are shown in Fig 1.

Criterion heart rate: the criterion measure was obtained using the Polar H7 heart rate monitor (Polar Electro, Kempele, Finland), which contains a single flexible plastic sensor (2.4 x 27.9 cm), worn concurrently and placed on the sternum. The Polar H7 heart rate receiver has a sample rate of 1000 Hertz and has high agreement with ECG measurements during various exercise modalities (treadmill, cycle, elliptical; Lins’ concordance correlation coefficient = 0.99 each) [11].

ECG chest strap device and watch: Suunto Spartan Sport watch (Suunto Oy, Vantaa, Finland)—the watch was secured around the right wrist by the strap. Heart rate was obtained through the accompanying heart rate strap that was secured around the sternum just below the Polar H7 monitor. The dimensions are 50 x 50 x 13.8 mm.

Photoplethysmography-based devices: Garmin Fenix 5 wristwatch (Garmin Ltd, Olathe, KS)—the device was secured to the left wrist by the strap. This device utilizes the Garmin Elevate^™^ multi-sensor heart rate monitor. The dimensions are 47 x 47 x 15.5 mm.

Jabra Elite Sport earbuds (Jabra, Copenhagen, Denmark)—earbuds were secured into the ear canal. The dimensions are 120 x 45 x 179 mm.

Motiv ring (Motiv Inc, San Francisco, CA)—a size 10 ring was secured on the finger that provided the best fit, being able to fit over the knuckle and fit snugly on the finger between the proximal interphalangeal and metacarpophalangeal joints. The appropriate finger was self-selected by each participant. The outer housing is composed of a titanium alloy, and employs a green LED optical heart rate sensor.

Scosche Rhythm+ forearm band (Scosche Industries Inc., Oxnard, CA)—secured around the forearm by a breathable neoprene band. Sensor size is 54.4 x 48.8 x 14.7 mm.

Heart rate data for the Polar H7, Rhythm+ forearm band, Jabra Elite earbuds, and Suunto Spartan Sport were transmitted real time via Bluetooth to a synced iPad mini tablet (Apple Inc., Cupertino, CA) and captured into the PerformTek application (Valencell Inc., Raleigh, NC). The PerformTek application reports heart rate in a second-by-second fashion and allowed the Rhythm+ forearm band, Jabra Elite earbuds, and Suunto Spartan Sport to be time-synced with the Polar H7. Heart rate from the Motiv ring was transmitted via Bluetooth to the synced iPad mini and captured into the Motiv application (Motiv Inc, San Francisco, CA). Heart rate data from the Garmin Fenix 5 was captured onto the watch during the trail run and synced to the Garmin Connect application on the iPad mini immediately upon return to the trailhead. Heart rate data from the Motiv ring and Garmin Fenix 5 were plotted against the criterion measure to visually confirm time-syncing between devices.

### Statistical analysis

All data are expressed as mean±SD. Except for the Motiv ring (minute-by-minute), data analysis was performed on second-by-second values expressed in beats per minute (bpm). Validity was determined through multiple methods [6]: Bland-Altman bias and Limits of Agreement (LOA) for repeated samples with accompanying 95% confidence intervals (CI) [33], Mean Absolute Error (MAE) and Mean Absolute Percent Error (MAPE), Lin’s Concordance Coefficient (r_C_), and Intraclass Correlations (ICC) (IBM SPSS, IBM Statistics version 24.0, Armonk, NY). Validity was considered if devices met all of the following criteria: MAPE within 5% [7], r_C_ greater than 0.90 [8, 34], and ICC greater than 0.70 with a p-value less than 0.05 [35].

## Results

**Fig 2:**
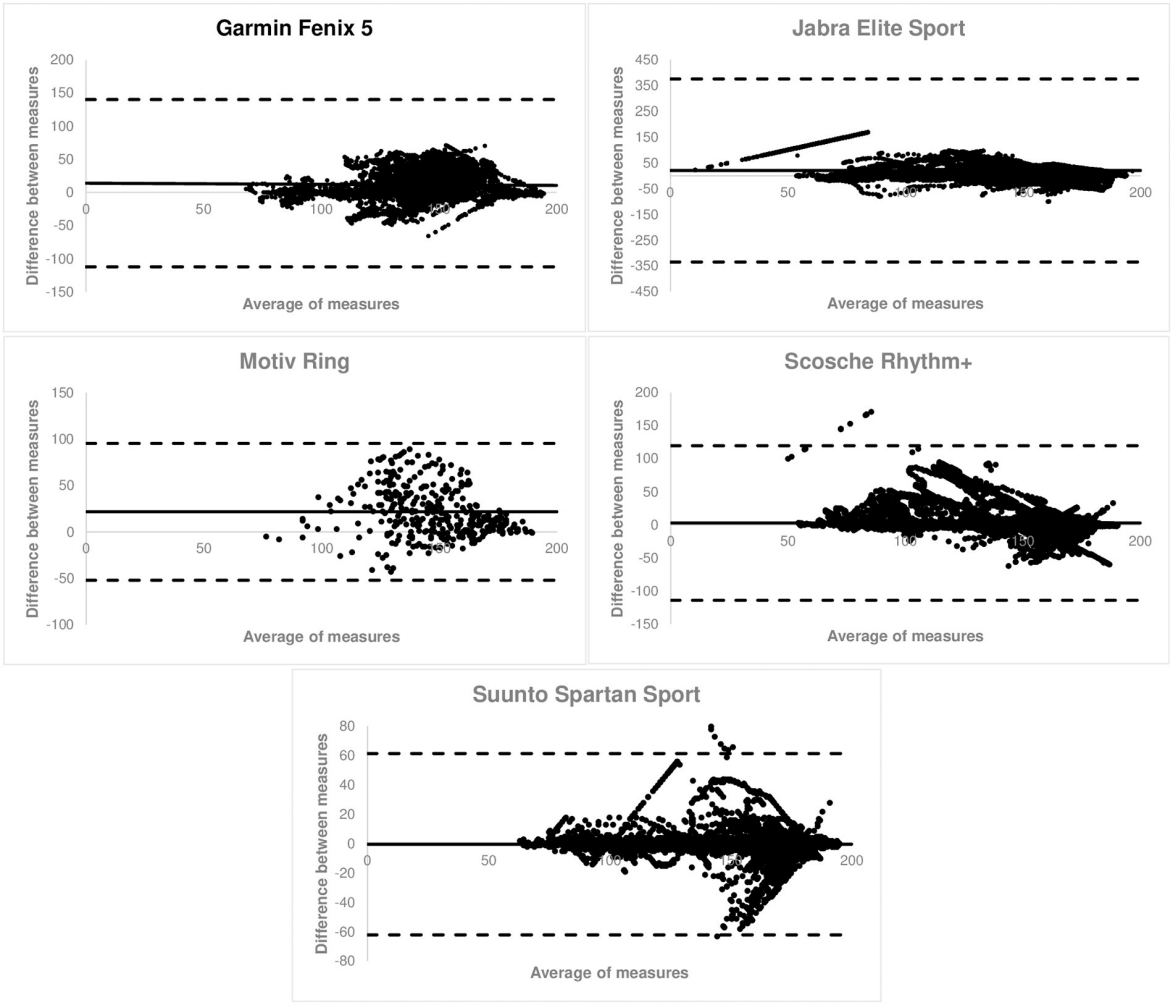
Bland-Altman plots pooled for all participants for the entire trail run by device.

The average time of the 2-mile trail run was 21:56 (5:38) min (mean [SD]). The uphill portion of the run was 11:39 (3:19) min, and the downhill portion was 10:26 (2:17) min. Validity measures for heart rate (bpm) over the entire length of trail run are shown in Table 1. Heart rate (bpm) validity for the uphill portion is shown in Table 2, and the return is displayed in Table 3. Bland Altman plots for each device for the entire trail run are displayed in Fig 2.

**Table 1:** Validity measures from participants (N = 21) who wore a concurrent criterion device during a 2-mile trail run.

	Garmin Fenix 5	Jabra Elite Sport	Motiv Ring	Scosche Rhythm+	Suunto Spartan Sport
Heart rate (bpm)	143 (24)	126 (58)	137 (27)	149 (29)	157 (24)
MAPE (%)	13.5	21.3	15.9	5.6	1.9
MAE (bpm)	20.8	30.0	25.1	7.3	2.9
Bias (SD)	15.9 (473.8)	19.2 (1119.6)	21.7 (488.8)	2.9 (74.1)	-0.3 (9.3)
Limits of Agreement (95% CI)	-32.1 (-140.8 to -123.5) to 162.1 (153.4 to 170.7)	-464.1 (-475.8 to -452.4) to 502.6 (490.9 to 514.2)	-52.0 (-62.3 to -41.8) to 95.5 (85.2 to 105.7)	-113.9 (-117.4 to -110.5) to 119.6 (116.2 to 123.1)	-62.1 (-63.5 to -60.7) to 61.4 (60.1 to 62.8)
r_C_ (95% CI)	0.316 (0.305 to (0.326)	0.384 (0.377 to 0.390)	0.293 (0.225 to 0.358)	0.780 (0.774 to 0.786)	0.955 (0.953 to 0.956)
ICC (p-value)	0.415 (<0.001)	0.395 (<0.001)	0.287 (<0.001)	0.120 (<0.001)	0.955 (<0.001)

Data expressed as mean (SD). MAPE = Mean absolute percent error, MAE = Mean Absolute Error, r_C_ = Lin’s Concordance Correlation Coefficient, ICC = Intraclass correlation coefficient.

**Table 2:** Validity measures from participants (N = 21) who wore a concurrent criterion device during the 1-mile uphill portion of a 2-mile trail run.

	Garmin Fenix 5	Jabra Elite Sport	Motiv Ring	Scosche Rhythm+	Suunto Spartan Sport
Heart rate (bpm)	134 (20)	125 (63)	134 (29)	146 (32)	154 (23)
MAPE (%)	13.7	24.5	16.4	6.2	1.5
MAE (bpm)	21.7	35.9	25.6	9.3	2.1
Bias (SD)	19.4 (203.8)	21.6 (1223.1)	22.6 (351.1)	3.9 (120.0)	-0.1 (2.0)
Limits of Agreement (95% CI)	-65.6 (-74.4 to -56.8) to 104.3 (95.5 to 113.1)	-254.8 (-271.5 to -238.2) to 298.1 (281.4 to 314.7)	-231.4 (-261.0 to -201.8) to 276.7 (247.1 to 306.3)	-88.4 (-94.6 to -82.2) to 96.1 (89.9 to 102.3)	-34.7 (-35.8 to -33.7) to 34.9 (33.8 to 35.9)
r_C_ (95% CI)	0.450 (0.435 to 0.465)	0.314 (0.304 to 0.323)	0.394 (0.305 to 0.476)	0.699 (0.689 to 0.709)	0.964 (0.962 to 0.966)
ICC (p-value)	0.450 (<0.001)	0.371 (<0.001)	0.393 (<0.001)	0.065 (<0.001)	0.955 (<0.001)

Data expressed as mean (SD). MAPE = Mean absolute percent error, MAE = Mean Absolute Error, r_C_ = Lin’s Concordance Correlation Coefficient, ICC = Intraclass correlation coefficient.

**Table 3:** Validity measures from participants (N = 21) who wore a concurrent criterion device during the 1-mile downhill return of a 2-mile trail run.

	Garmin Fenix 5	Jabra Elite Sport	Motiv Ring	Scosche Rhythm+	Suunto Spartan Sport
Heart rate (bpm)	142 (21)	135 (56)	141 (25)	152 (26)	158 (24)
MAPE (%)	13.4	20.6	15.4	3.8	2.2
MAE (bpm)	20.0	29.5	24.7	5.4	3.4
Bias (SD)	11.6 (258.3)	16.6 (991.2)	20.9 (603.4)	1.9 (29.3)	-0.6 (13.6)
Limits of Agreement (95% CI)	-92.5 (-101.0 to -83.9) to 115.8 (107.2 to 124.3)	-214.3 (-230.1 to -198.4) to 247.4 (231.5 to 263.3)	-133.7 (-153.3 to -114.2) to 175.5 (155.9 to 195.0)	-67.7 (-70.6 to -64.7) to 71.5 (68.5 to 74.5)	-50.6 (-52.6 to -48.6) to 49.4 (47.4 to 51.5)
r_C_ (95% CI)	0.381 (0.361 to 0.400)	0.397 (0.386 to 0.407)	0.157 (0.054 to 0.256)	0.885 (0.880 to 0.889)	0.949 (0.947 to 0.951)
ICC (p-value)	0.381 (<0.001)	0.431 (<0.001)	0.145 (<0.001)	0.885 (<0.001)	0.949 (<0.001)

Data expressed as mean (SD). MAPE = Mean absolute percent error, MAE = Mean Absolute Error, r_C_ = Lin’s Concordance Correlation Coefficient, ICC = Intraclass correlation coefficient.

## Discussion

The purpose of this investigation was to evaluate heart rate validity of several types of wearable technology devices during a variable intensity trail run. We hypothesized that all devices would have acceptable heart rate agreement compared to our criterion over the course of this exercise bout. The main findings are that regardless of device location (finger, wrist, ear, forearm), PPG-based devices do not provide acceptable heart rate validity during a trail run lasting longer than 20-min. We found that a heart rate strap recorded to a wrist watch (Suunto Spartan Sport), provided acceptable agreement for heart rate utilizing thresholds normally applied to laboratory-based research.

The Suunto Spartan Sport device has been evaluated with respect to step count accuracy [36], and proposed as a wearable capable of returning the cardiorespiratory fitness component of an integrated cross-modal cybernetic health status assessment [37]. The device has also been utilized in an outdoor environment to track altitude profile during a 64 km ultra-endurance race [38] and Grand Canyon rim to rim hike [39], and pacing and stride variations during a 44 km trail run performed in tropical conditions [40]. To our knowledge, concurrent heart rate validity has not been determined compared to a criterion measure. Thus, we report for the first time that when paired with accompanying heart rate strap, the Suunto Spartan Sport displays acceptable heart rate validity during variable intensity trail running. As the heart rate strap is an ECG-based wearable similar to the criterion measure (Polar H7) utilized in the current study, these findings should be expected.

Forearm PPG heart rate monitors have been utilized in a variety of applications including virtual reality ship handling simulators [41], during an interactive game of tag [42], evaluating driving during various weather conditions [43], and for remote monitoring of triathlon training [44]. Two investigations have determined heart rate validity of forearm devices during exercise [11, 22]. The Scosche Rhythm+ heart rate agreed with the ECG criterion at rest (r_C_ = 0.93), and during cycle and treadmill exercise lasting 4.5 min (r_C_ = 0.84, 0.92), but not when performing elliptical training (r_C_ = 0.41 with arms, 0.27 without arm movement) [11]. Another investigation reported no difference in heart rate compared to Polar H7 measures when participants completed 5-min of walking and running both on a treadmill and in an unrestricted setting (p>0.05) [22]. The current investigation extends the literature on forearm heart rate monitors to outdoor exercise in the form of variable intensity trail running. Our results are interesting in this regard, as better agreement was observed during downhill running (MAPE = 3.8%, bias = 1.9 bpm, r_C_ = 0.885) than when running was at a generally positive incline (MAPE = 6.2%, bias = 3.9 bpm, r_C_ = 0.699). It is possible that greater device motion was experienced in the uphill portion leading to these results, and further investigation is warranted.

The Garmin Fenix series is relatively new PPG-based device and as such has limited available literature. In fact, we were only able to find two conference abstracts for the Fenix 3 [45, 46], and one recent laboratory-based investigation on the Fenix 5 [47]. The Garmin Fenix 3 estimation of maximal aerobic capacity was not different compared to laboratory-based metabolic analysis (p>0.05) [46]. Biomechanical running parameters of stride length and run cadence were not different compared to laboratory measurements (p>0.05), but significant differences were observed for vertical oscillation and ground contact time (p<0.05) [45]. Düking et al. reported moderate heart rate validity for the Garmin Fenix 5 during sitting and walking (standardized typical error of the estimate [sTEE] = 0.63, 0.62)to be poor with increased intensities of exercise (9.9 MET level sTEE = 1.24, 13.8 MET level = 1.44) and recommend caution due to the higher rates of error [47]. The results of the current investigation extend the literature in that poor heart rate validity measures in the Garmin Fenix 5 were observed during a variable intensity trail run (MAPE = 13.5%, bias = 15.9 bpm, r_C_ = 0.316), and these measures are consistent regardless of the incline (either primarily uphill or downhill) of the trail.

While ring-based wearable devices have been presented in conference abstracts [48–51], we have been unable to find published literature incorporating its use during exercise. Thus, the current investigation reports concurrent heart rate validity in a PPG ring-based wearable device during exercise for the first time. We must recommend caution for this type of device, as heart rate validity during trail running exercise was the poorest of all PPG devices tested (MAPE = 15.9%, bias = 21.7 bpm, r_C_ = 0.293). Future investigations utilizing ring-based wearables should be investigated in a controlled laboratory setting to determine heart rate validity in this environment. A limitation with respect to this device is that the current investigation utilized a single ring size. Additionally, the specific fingers utilized by participants was not recorded and future studies should take this into account.

Literature on PPG-based earbud heart rate validity during exercise is beginning to emerge. Investigations have found earbud-based heart rate to be acceptable during resistance training (MAPE = 6.24%) [9], graded exercise testing on a treadmill (bias = -0.2%, R^2^ = 0.98) [52], and during treadmill exercise and high intensity training exercises (bias = 0.8 bpm, MAPE = 2.48%, r_C_ = 0.943; bias = -3.6 bpm, MAPE = 3.53%, r_C_ = 0.861 respectively) [8]. When utilized in patients with cardiac diseases, these devices tended to have less agreement when heart rate was above 100 bpm and in participants with atrial fibrillation (average difference to true heart rate = 20.3 bpm, r^2^ = 0.434) [53]. One investigation utilizing cycle graded exercise testing found that heart validity decreased in earbud devices as the intensity of this exercise increased (MAPE at 50W = 6.4%, MAPE at 200W = 15.42%) [9]. The results of the current study indicate that heart rate validity in PPG-based earbud devices is poor during running in an outdoor environment at variable intensities of exercise.

The current investigation utilized a variety of commercially available wearable technology devices capable of returning heart rate measures. We observed a range of heart rate agreement in devices during a trail run and as expected found an ECG-based chest strap recording to a wrist worn watch to have the greatest concurrent validity. The PPG-based devices were positioned at various locations on the body, and the forearm strap device displayed the best validity of this class of wearable. Nevertheless, in an outdoor environment, heart rate obtained from the remaining PPG-based devices (wrist, forearm, ring-based, and earbuds) were observed to have poor agreement with the criterion measure. Motion artifact is the likely source of error in wearable devices that were found to have low heart rate validity [23], however the influence of ambient light [54] should also be considered another source of potential error, particularly with the use of photoplethysmography-reliant devices during outdoor exercise. The Consumer Technology Association recommends 5-min exercise durations when validating wearable technology devices [55], and investigations specific to running have utilized protocols of 1.5-minutes [11], 3-minutes [14, 15, 18, 19], 4-minutes [17], 5-minutes [4, 7, 8, 10], and 6-minutes [16]. The current investigation is unique in that heart rate validity was assessed over a much longer duration (average running time was near 22 min). Toward this end we recommend that future studies continue to determine validity in applied settings and over longer time periods that are more in line with durations being utilized by exercising individuals.

Laboratory-based research investigations into wearable technology devices have generally utilized specific thresholds for validity measurements, such as ICC above 0.70 and/or MAPE lower than 5% [7]. While these thresholds are acceptable for controlled environments, wearable devices are being used in a variety of applications that do not offer the same constraints. While we believe that researchers and consumers can make their own determination regarding the margin of error that is acceptable to their specific application, the findings of the current investigation indicate that PPG-based devices, regardless of location on the body, display poor heart rate validity during variable intensity running in a natural environment. Until technological advances occur in PPG-based devices allowing for acceptable agreement, heart rate in outdoor environments should be obtained using an ECG-based chest strap that can be connected to a wristwatch or other comparable receiver.
